# (−)-Epigallocatechin gallate inhibits endotoxin-induced expression of inflammatory cytokines in human cerebral microvascular endothelial cells

**DOI:** 10.1186/1742-2094-9-161

**Published:** 2012-07-06

**Authors:** Jieliang Li, Li Ye, Xu Wang, Jinping Liu, Yizhong Wang, Yu Zhou, Wenzhe Ho

**Affiliations:** 1 The Center for Animal Experiment/Animal Biosafety Level III Laboratory, Wuhan University Wuhan, Hubei, 430071, People's Republic of China; 2 Department of Pathology and Laboratory Medicine, Temple University School of Medicine, 843 MERB, 3500N Broad Street, Philadelphia, PA, 19140, USA

**Keywords:** 67LR, endothelial, (−)-epigallocatechin gallate, LPS, NF-κB

## Abstract

**Background:**

(−)-Epigallocatechin gallate (EGCG) is a major polyphenol component of green tea that has antioxidant activities. Lipopolysaccharide (LPS) induces inflammatory cytokine production and impairs blood–brain barrier (BBB) integrity. We examined the effect of EGCG on LPS-induced expression of the inflammatory cytokines in human cerebral microvascular endothelial cells (hCMECs) and BBB permeability.

**Methods:**

The expression of TNF-α, IL-1β and monocyte chemotactic protein-1 (MCP-1/CCL2) was determined by quantitative real time PCR (qRT-PCR) and ELISA. Intercellular adhesion molecule 1 (ICAM-1) and vascular cell adhesion molecule (VCAM) in hCMECs were examined by qRT-PCR and Western blotting. Monocytes that adhered to LPS-stimulated endothelial cells were measured by monocyte adhesion assay. Tight junctional factors were detected by qRT-PCR (Claudin 5 and Occludin) and immunofluorescence staining (Claudin 5 and ZO-1). The permeability of the hCMEC monolayer was determined by fluorescence spectrophotometry of transmembrane fluorescin and transendothelial electrical resistance (TEER). NF-kB activation was measured by luciferase assay.

**Results:**

EGCG significantly suppressed the LPS-induced expression of IL-1β and TNF-α in hCMECs. EGCG also inhibited the expression of MCP-1/CCL2, VCAM-1 and ICAM-1. Functional analysis showed that EGCG induced the expression of tight junction proteins (Occludin and Claudin-5) in hCMECs. Investigation of the mechanism showed that EGCG had the ability to inhibit LPS-mediated NF-κB activation. In addition, 67-kD laminin receptor was involved in the anti-inflammatory effect of EGCG.

**Conclusions:**

Our results demonstrated that LPS induced inflammatory cytokine production in hCMECs, which could be attenuated by EGCG. These data indicate that EGCG has a therapeutic potential for endotoxin-mediated endothelial inflammation.

## Background

The brain endothelial cells interact with resident cells in the central nervous system (CNS), providing the protective blood–brain barrier (BBB) interface between the CNS and peripheral blood system. By controlling the access of blood components, including immune cells to the CNS, the BBB regulates the delicate milieu optimal for neuronal communication and helps to maintain the homeostasis of the CNS. Breakdown of BBB is an early and significant event in CNS inflammation induced by extrinsic or intrinsic stimuli, including endotoxins [1]. It has been shown that brain endothelial cells are a primary target of immunological attack in bacterial infection, and their injury can lead to vascupathy and organ dysfunction associated with disruption of tight junctions in the brain endothelium [2].

Lipopolysaccharide (LPS), a product of bacterial infection, is known to induce inflammatory cytokines and impairs the BBB system. A central feature of the pathophysiology of acute inflammation and septic shock triggered by LPS is the production of multiple proinflammatory mediators such as cellular adhesion molecules, cytokines, and chemokines by monocytes–macrophages as well as vascular endothelial cells [3]. LPS can directly elicit a variety of inflammatory cytokines in endothelial cells, such as inducible nitric oxide synthase (iNOS) [4], IL-1β [5] and IL-6 [6,7]. Furthermore, LPS, TNF-α, or IL-1β can significantly stimulate human brain microvascular endothelial cells (HBMECs) to release monocyte chemotactic chemotacticprotein-1 (MCP-1/CCL2), an important factor for monocyte migration bound to the apical endothelial surfaces [8].

Intact cerebral endothelial cells constitutively express low levels of intercellular adhesion molecule 1 (ICAM-1), which plays a vital role in the process of leukocyte transmigration through endothelial cell barriers and has been shown to mediate signal transduction events in endothelial cells induced either by its cross-linking or by the binding of T lymphocytes [9]. However, when stimulated by LPS or certain cytokines, these cells produce high levels of ICAM-1 [10,11]. The cell walls of *Streptococcus pneumoniae* (PCW), the most common cause of adult bacterial meningitis, also induces ICAM-1 expression in rat primary brain microvascular endothelial cell cultures [4]. This induction could be completely blocked by TNF-α antibody, suggesting that ICAM-1 expression is mediated by the inflammatory cytokine and that cerebral endothelial cells regulate critical steps in inflammatory BBB disruption of bacterial meningitis. Therefore, regulation of endothelial adhesion molecule and cytokine expression in brain endothelial cells is critical in maintaining BBB integrity.

(−)-Epigallocatechin gallate (EGCG), also known as epigallocatechin 3-gallate, is the most abundant catechin in green tea. EGCG as a potent antioxidant has been shown to have both anti-inflammatory and anti-atherogenic properties in experimental studies conducted *in vitro* and *in vivo*[12,13]. EGCG was found to inhibit TNF-α-induced production of MCP-1/CCL2 from bovine coronary artery endothelial cells, providing direct vascular benefits in inflammatory cardiovascular diseases [14]. Previous studies have also demonstrated that EGCG attenuated the increase in malondialdehyde levels caused by cerebral ischemia and reduced the formation of post-ischemic brain edema and infarct volume [15]. This study suggests that EGCG is a neuroprotective agent against excitotoxicity-related neurologic disorders such as brain ischemia. Further, the neuroprotective effect of EGCG against ischemia-induced brain damage was found, in part, to be due to modulation of NOS isoforms and preservation of mitochondrial complex activity and integrity [16]. Thus, the *in vivo* neuroprotective effects of EGCG are not exclusively due to its antioxidant effects but involve more complex signal transduction mechanisms. In this study, we examined whether EGCG possesses the ability to protect the endothelial monolayer from loss of tight junction proteins and disruption of BBB permeability. We used a well-established *in vitro* model of the human BBB to monitor the effects of LPS and/or EGCG on the inflammatory responses and barrier permeability. We also investigated the mechanisms through which EGCG exerts its action on LPS-induced inflammation in human brain endothelial cells.

## Materials and methods

### (−)-Epigallocatechin gallate

EGCG (≥95%) was purchased from Sigma-Aldrich St. Louis, MO**,** USA (CAS#: 989-51-5; Cat# E4143). EGCG stock solution was prepared in sterile double distilled water at 20 mM.

### Brain endothelial cell culture

Human cerebral microvascular endothelial cell (hCMEC) line, D3 clone, was developed by immortalization of the primary human brain vascular endothelial cells after transduction with lentiviral vectors encoding the catalytic subunit of human telomerase hTERT and SV40 T antigen, as described previously [17]. The hCMEC/D3 cell line recapitulates most of the unique properties of brain endothelium and may thus constitute a well established *in vitro* model of the human BBB [17]. It has also been reported that D3 cell line maintains the *in vitro* physiological permeability barrier properties of the BBB, even in the absence of abluminal astrocytes [18]. D3 cells express typical endothelial markers (VE-cadherin and Occudin) and have been successfully used as an *in vitro* model of the BBB [19-21]. D3 cells were grown in endothelial cell growth medium (EGM®; Lonza, Walkersville, MD, USA) supplemented with vascular endothelial growth factor, insulin-like growth factor-1, epidermal growth factor, basic fibroblast growth factor, Gentamicin, ascorbic acid, heparin, fetal bovine serum and hydrocortisone (Lonza, Walkersville, MD, USA). Cells were cultured on collagen-coated (BD Biosciences,Rockville, IL, USA) tissue culture plate in a humidified atmosphere at 37°C in 5% CO_2_.

### Treatment of endothelial cells

hCMEC/D3 cells were treated with 100 ng/ml LPS for different time periods (3, 6, 24, and 48 h) or with different concentrations of LPS (1, 10, 100, and 1000 ng/ml) for 6 h. For the pretreatment, EGCG was added to the culture media (1, 5, and 25 μM) 1 h prior to LPS treatment and further incubated together for 6 h. To test the blockage effect of 67-kDa laminin receptor (67LR), cells were incubated with mouse monoclonal antibody against LR (clone MluC5; 5 μg/ml; NeoMarkers, Fermont, CA, USA) or isotype control mouse IgM for 1 h before the addition of EGCG to the cell cultures. To test the direct effect of EGCG on the expression of Toll-like receptor (TLR)4 and Myeloid differentiation primary response gene (88) (MyD88), hCMEC/D3 cells were treated with 5 μM of EGCG for 12 to 72 h or with 0 to 25 μM of EGCG for 24 h.

### Reverse transcription and quantitative real time PCR

Total RNA was extracted with Tri-reagent (Sigma-Aldrich) and quantitated by spectrophotometric analysis. Reverse transcription was performed using the AMV transcriptase and RNasin (Promega Co., Madison, WI, USA) according to the manufacturer’s instruction. Quantitative real time PCR (qRT-PCR) was performed with Brilliant SYBR Green Master Mix (Bio-Rad Laboratories, Hercules, CA, USA) described previously [22]. The primers that were used for the PCR amplifications are listed in Table 1. The oligonucleotide primers were synthesized by Integrated DNA Technologies, Inc. (Coralville, IA, USA). All values were calculated using the delta delta Ct method and expressed as the change relative to the expression of glyceraldehyde 3-phosphate dehydrogenase (GAPDH) mRNA.

**Table 1:** Primer sequences for the quantitative real time PCR

**Gene name**	**Forward**	**Reverse**
**TNF-**α	5’-CGA GTG ACA AGC CTG TAG C-3’	5’-GGT GTG GGT GAG GAG CAC AT-3’
**IL-1**β	5’-AAG CTG ATG GCC CTA AAC AG-3’	5’-AGG TGC ATC GTG CAC ATA AG-3’
**MCP-1/CCL2**	5’-CAT AGC AGC CAC CTT CAT TCC-3’	5’-TCT GCA CTG AGA TCT TCC TAT TGG-3’
**ICAM-1**	5’-CCT TCC TCA CCG TGT ACT GG-3’	5’-AGC GTA GGG TAA GGT TCT TGC-3’
**VCAM-1**	5’-GGG AGC TCT GTC ACT GTA AG-3’	5’-ATC CGT ATC CTC CAA AAA CT-3’
**Occludin**	5’-AAG CAA GTG AAG GGA TCT GC-3’	5’-GGG GTT ATG GTC CAA AGT CA-3’
**Claudin 5**	5’-GTC TTT ACT CCA TCG GCA GG-3’	5’-TTT TTT TTT TTT GAG AGT TCA AAC C-3’
**TLR4**	5’-CAT TGC TTC TTG CTA AAT GCT G-3’	5’-GGA TTA AAG CTC AGG TCC AGG-3’
**MyD88**	5’-CCG CGC TGG CGG AGG AGA TGG AC-3’	5’-GCA GAT GAA GGC ATC GAA ACG CTC-3’
**GAPDH**	5’-GGT GGT CTC CTC TGA CTT CAA CA-3’	5’-GTT GCT GTA GCC AAA TTC GTT GT-3’

GAPDH, glyceraldehyde 3-phosphate dehydrogenase; ICAM, intercellular adhesion molecule; MCP, monocyte chemotactic protein; TLR, Toll-like receptor; VCAM, vascular adhesion molecule.

### ELISA

**Figure 1:**
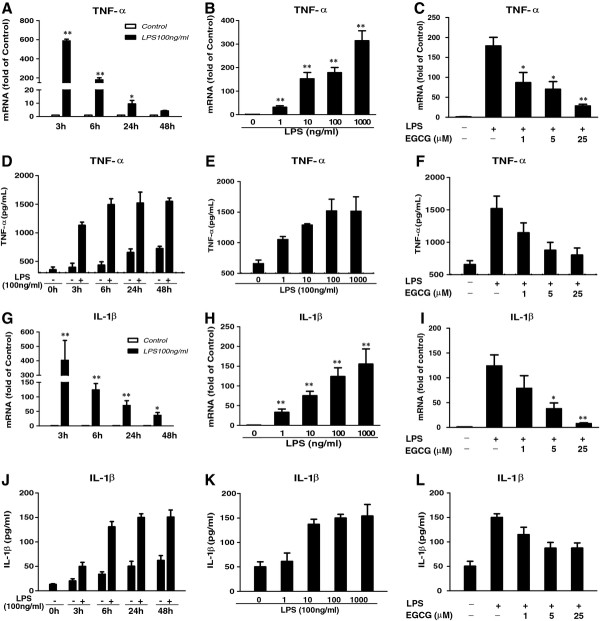
**Effect of (−)-epigallocatechin gallate on lipopolysaccharide-induced inflammatory cytokine expression at both mRNA and protein levels.** Human cerebral microvascular endothelial cells/D3 (hCMEC/D3) were treated with or without lipopolysaccharide (LPS) at 100 ng/mL for the indicated time periods (**A**,**D**,**G**,**J**) or at indicated concentrations for 6 h (**B**,**H**) or 24 h (**E**,**K**). (**C**,**F**,**I** and **L**) hCMEC/D3 cells were pretreated with (−)-epigallocatechin gallate (EGCG) at the indicated concentrations for 1 h prior to LPS treatment (100 ng/mL) for an additional 6 h (C,I) or 24 h (F,L). The mRNA expression was determined by quantitative real time PCR, and protein levels in the culture medium were measured by conventional ELISA. Data are expressed as mean ± SD of three different experiments (*P < 0.05, **P < 0.01, compared with no treatment control or LPS treatment only).

**Figure 2:**
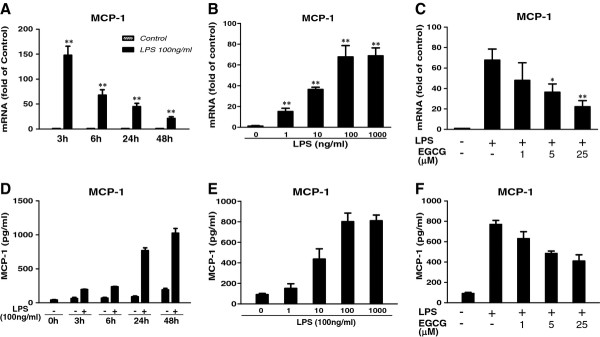
**Effect of (−)-epigallocatechin gallate on lipopolysaccharide-induced MCP-1 expression.** Human cerebral microvascular endothelial cells/D3 (hCMEC/D3) were treated with 100 ng/mL of lipopolysaccharide (LPS) for the indicated time periods (**A**,**D**) or with LPS at the indicated concentrations for 6 h (**B**) or 24 h (**E**). (**C**,**F**) hCMEC/D3 cells were pretreated with (−)-epigallocatechin gallate (EGCG) at the indicated concentrations for 1 h prior to LPS treatment (100 ng/mL) for an additional 6 h (C) or 24 h (F). The mRNA expression was determined by quantitative real time PCR and protein levels in the culture medium were measured by conventional ELISA. Data are expressed as mean ± SD of three different experiments (*P < 0.05, **P < 0.01, compared with no treatment control or LPS treatment only).

TNF-α, IL-1β and MCP-1/CCL2 gene expressions, identified from RT-PCR, were evaluated for protein expression using ELISA. After hCMEC/D3 cells were treated as indicated in the figure Figure. 1 and 2, conditioned medium was collected and levels of TNF-α, IL-1β and MCP-1/CCL2 were measured using conventional double sandwich ELISA kits from eBioscience Inc. (San Diego, CA, USA). Assays were performed according to the manufacturer's instructions.

### Immunofluorescence assay

hCMEC/D3 cells were cultured on chamber slides or glass coverslips in 24-well plate coated with collagen. After treatment, cells were washed with ice-cold PBS (with Ca^2+^ and Mg^2+^) twice, then fixed at 4°C in 4% paraformaldehyde plus 4% sucrose in PBS for 30 minutes. Subsequently, the cells are permeated with 0.2% Triton X-100 in PBS on ice for an additional 10 minutes. Cells were blocked in Block Solution (Pierce, Rockford, IL, USA) for 1 h at room temperature. The cells were then incubated at room temperature with mouse anti-Claudin 5 (1:50; Invitrogen, Grand Island, NY, USA) or mouse anti-ZO-1 (1:50; Invitrogen) for 1 h. After three washes with PBS, cells were incubated with Alexa488-conjugated goat anti-mouse IgG (1:250) for 1 h. Cells were then viewed under a fluorescence microscope (Olympus IX71, Japan).

### Western blot analysis

The expression of the tight junctional proteins Claudin-5 and Occludin in hCMEC/D3 was evaluated by immunoblot analysis. Following incubation with specific antibodies and extensive washing in PBS containing 0.05% Tween-20, membranes were incubated with horseradish peroxidase-conjugated goat antimouse IgG (Pierce, Chester, UK) for 1 h at room temperature. Membranes were extensively washed in PBS containing 0.05% Tween-20, and immunoblots were visualized by enhanced chemiluminescence detection (ECL, Amersham, Bucks, UK).

### Monocyte adhesion assay

Endothelial hCMEC/D3 cells were plated at 10^4^cells/well in 200 μl clonetics medium in collagen-coated black bottom 96-well plates for 3 days. Cell cultures were then maintained in partial clonetics medium (without growth factors) for an additional 72 h prior to the treatment with LPS and/or EGCG. Monocytes were isolated from peripheral blood mononuclear cells of a healthy donor as described previously [23] at the Path Bio Resource Human Immunology Core in accordance with protocols approved by the Human Subjects Research Committee of the University of Pennsylvania. The purity of isolated monocytes was higher than 95%. Cell viability was determined by trypan blue exclusion assay and monocyte viability above 95% was used. Briefly, monocytes were resuspended in serum-free DMEM at 5 **×** 10^6^ cells/ml and labeled with 5 μM Calcein-AM (Molecular Probes, OR, USA) at 37°C for 30 minutes. After washing with serum-free medium twice, the monocytes were resuspended in clonetics medium at 10^6^ cells/ml. The labeled monocytes (10^5^ cells) were then added to the endothelial hCMEC/D3 monolayer and incubated for 15 minutes at 37°C to allow the adhesion [24]. The plate was then carefully rinsed twice with 1**×** PBS to remove non-adherent monocytes and read on a fluorescent plate reader at 494/517 nm. The actual number of adherent monocytes was determined by comparing a standard plate with known numbers of labeled monocytes.

### Transendothelial diffusion

The endothelial cell permeability was assessed by determining the flux of fluorescein through the hCMEC/D3 monolayer using a procedure described elsewhere with a slight modification [25,26]. Briefly, hCMEC/D3 cells were plated onto collagen-coated 0.4-μm Millicell hanging cell culture inserts (Millipore, MA, USA) and left to reach confluence for 7 to 10 days with a culture media change every 2 to 3 days. Cells were then treated with LPS in the presence or absence of EGCG for 24 h. After treatment, fluorescein sodium salt in clonetics medium was loaded to the apical filter compartment to produce an initial concentration of 1 μM. Subsequently, 100 μl medium was removed from the basolateral compartment after 60 minutes. The fluorescence was measured with a fluorescence microplate reader (PerkinElmer 1420 Multilabel Counter, Bridgeville, PA, USA) at 488/525 nm. The concentration of fluorescein sodium salt in the lower compartment represents the endothelial monolayer permeability [26].

### Transendothelial electrical resistance

To determine the integrity of brain endothelial monolayers, transendothelial electrical resistance (TEER) measurements were performed using the 1600R ECIS system (Applied Biophysics, Troy, NY, USA). The ECIS system provides real-time monitoring of changes in TEER. Briefly, hCMEC/D3 at 10^5^ per well were plated on collagen type I-coated 96W10E + electrode arrays (Applied Biophysics). The cells were then allowed to form monolayers reaching stable TEER values. After 4 days (with a media change every 2 days), the monolayers were exposed to various concentrations of LPS with or without EGCG pretreatment as indicated. The readings were acquired continuously for 72 h at 4000 Hz and at 30-minute intervals. Confluent hCMEC/D3 monolayers showed baseline TEER readings between 500 and 800 Ω/cm^2^. The data are shown as a percentage change of baseline TEER along with the standard error of the mean of condition replicates.

### Transfection and luciferase assays

The plasmid (pNF-κB-Luc) containing NF-κB promoter linked with a luciferase gene was developed by Dr Petrak [27]. Two copies of the mouse k light chain enhancer [28] were cloned into pBLCAT3 vector [29], and then the construct was modified by replacing the CAT reporter with the luciferase gene obtained from pGEM-Luc plasmid [27]. DNA was prepared by Miniprep techniques, according to the manufacturer’s instruction (Qiagen, Valencia, CA, USA) and used in the transfection experiments. For each transfection experiment, the hCMEC/D3 cells were seeded in a 12-well tissue culture plate at a density of 3 × 10^5^ cells/well 1 day before transfection. The cells were transfected with the pNF-κB-Luc using FuGene HD Transfection Reagent (Roche Molecular Bilchemicals, Indianapolis, IN, USA) with a ratio of FuGene HD : plasmid 3:1 (μl:μg). Six hours after the transient transfection, the cells were incubated with or without EGCG (1, 5, and 25 μM) for 1 h, then treated with LPS for an additional 6 h. At the termination of the experiments, cells were washed twice with PBS, then lyzed in 0.1 ml 1× Cell Culture Lysis Buffer (Promega, Madison, WI, USA). Cell-free lysates were obtained by centrifugation at 13,000 × *g* for 2 minutes at 4°C. The effects of LPS/EGCG on the activation of NF-κB promoter in these transiently transfected cells were determined by NF-κB promoter-driven luciferase activity. Luciferase activity in cell lysate was quantified using a luciferase assay system (Promega) and a luminometer. The results were presented as relative light units and data were expressed as -fold of untreated cells. Measurements were performed by calculating the average of triplicate samples of two independent experiments.

### Statistically analysis

Data are expressed as the mean ± SD of at least three independent experiments. Statistical significance was analyzed using a one-way analysis of variance (ANOVA) followed by post Newman-Keul’s test. It was considered statistically significant when the *P*-value was less than 0.05.

## Results

### (−)-Epigallocatechin gallate inhibits lipopolysaccharide-mediated induction of inflammatory cytokine expression

We first investigated the effect of LPS on the inflammatory cytokine (TNF-α and IL-1β) expression in hCMEC/D3 cells. As shown in Figure 1, LPS treatment of hCMEC/D3 cells significantly induced the expression TNF-α and IL-1β at both mRNA (Figure 1A,B,G,H) and protein (Figure 1D,E,J,K) levels. The highest effect of LPS on these cytokines at the mRNA level was observed at 3 h post-treatment and diminished as time went by (Figure 1A,G). The induction of these cytokines at the mRNA and protein levels were both time- and dose-dependent (Figure 1B,D,E,H,J,K). EGCG pretreatment of hCMEC/D3 cells could compromise the induction effect of LPS on the cytokines at both the mRNA and protein levels (Figure 1C,F,I,L).

### (−)-Epigallocatechin gallate inhibits lipopolysaccharide-induced expression of monocyte chemotactic protein and adhesion molecule

**Figure 3:**
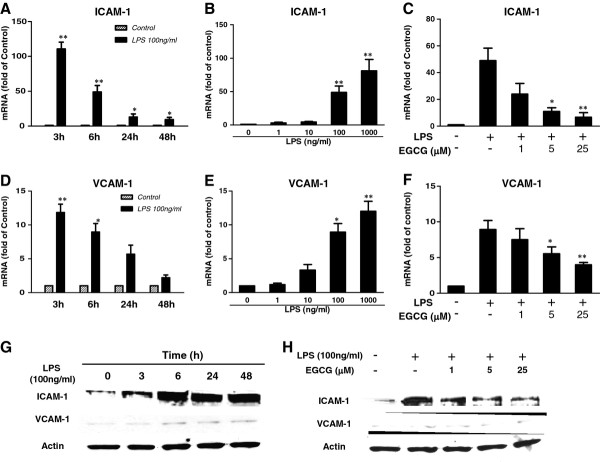
**Effect of (−)-epigallocatechin gallate on lipopolysaccharide-induced adhesion molecule expression of endothelial cells.** (**A**-**F**) Human cerebral microvascular endothelial cells/D3 (hCMEC/D3) were treated with 100 ng/ml of lipopolysaccharide (LPS) for the indicated time periods (3, 6, 24, 48 h) or with LPS at the indicated concentrations for 6 h. For pretreatment, cells were treated with (−)-epigallocatechin gallate (EGCG) (1, 5, and 25 μM) for 1 h prior to LPS treatment. The mRNA expression was determined by quantitative real time PCR. Data are expressed as mean ± SD of three different experiments (*P < 0.05, **P < 0.01, compared with no treatment control or LPS treatment only). (**G**) Cells were treated with 100 ng/ml of LPS for the indicated time periods and protein was extracted for western blotting of adhesion molecule expression. (**H**) hCMEC/D3 cells were treated with EGCG for 1 h prior to LPS treatment for 6 h. Protein was extracted for western blotting of adhesion molecule expression. Representative data from three independent experiments are shown.

Endothelial cells express MCP and adhesion molecules that mediate the interactions between cells of the immune system and the endothelium system. We found that LPS treatment of hCMEC/D3 cells induced the expression of MCP-1 in both time-dependent (Figure 2A,D) and dose-dependent (Figure 2B,E) fashions. EGCG pretreatment at 5 and 25 μM significantly inhibited the upregulation of MCP-1 by LPS treatment (Figure 2C,F). In addition, LPS could time- and dose-dependently induce the mRNA expressions of two adhesion molecules, ICAM-1 (Figure 3A,B) and vascular adhesion molecule (VCAM)-1 (Figure 3D,E). The induction of ICAM-1 and VCAM-1 by LPS was also observed at the protein level as determined by western blotting (Figure 3G). It was found that 6 h stimulation was enough to potently induce the protein expression of ICAM-1 and VCAM-1 (Figure 3G), with no significant increase when the treatment was extended to 24 h or 48 h. Therefore, we used 6 h stimulation when performing monocyte adhesion experiments. We next examined whether EGCG pretreatment of hCMECs could attenuate the expression of ICAM-1 and VCAM-1. We observed that EGCG pretreatment of hCMEC/D3 cells inhibited LPS-mediated induction of ICAM-1 and VCAM-1 at both the mRNA (Figure 3C,F) and protein levels (Figure 3H).

### (−)-Epigallocatechin gallate inhibits lipopolysaccharide-mediated monocyte adhesion to endothelial cells

**Figure 4:**
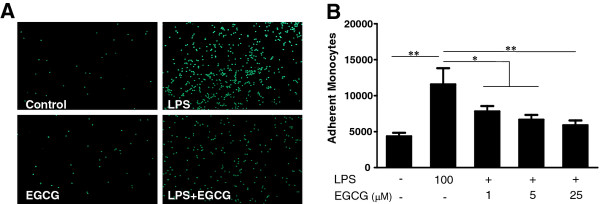
**Effect of (−)-epigallocatechin gallate on lipopolysaccharide-induced monocyte adhesion to endothelial cells.** (**A**) Human cerebral microvascular endothelial cells/D3 (hCMEC/D3) were pretreated with or without (−)-epigallocatechin gallate (EGCG) (25 μM) for 1 h prior to lipopolysaccharide (LPS) (100 ng/mL) treatment for an additional 6 h. Calcein-AM-labeled fresh monocytes were then added to hCMEC/D3 cell cultures and incubated for 15 minutes at 37°C. The fluorescence was measured at 494/517 nm and the actual numbers of adherent monocytes were determined by comparing a standard plate with known numbers of labeled monocytes. (**B**) hCMEC/D3 cells were treated with LPS at the indicated concentrations for 6 h, or EGCG (1, 5, 25 μM) for 7 h, or pretreated with EGCG (1, 5 or 25 μM) for 1 h followed by LPS treatment (100 ng/mL) for 6 h. Data are expressed as mean ± SD of three different experiments (*P < 0.05, **P < 0.01).

Since MCP-1, ICAM-1 and VCAM-1 are the key elements in mediating the adhesion of leukocytes to vascular endothelia, we investigated the effect of EGCG on monocyte adhesion to hCMEC/D3 cells. Figure 4A shows that LPS treatment of hCMEC/D3 significantly increased monocyte adhesion to the endothelial cell monolayer. This LPS effect was in a dose-dependent manner (Figure 4B). EGCG alone has little effect on monocyte adhesion whereas EGCG pretreatment of hCMEC/D3 cells attenuated LPS-induced adhesion of monocytes to the endothelial monolayer (Figure 4A,B).

### (−)-Epigallocatechin gallate blocks lipopolysaccharide-mediated suppression of Claudin 5 and Occludin

**Figure 5:**
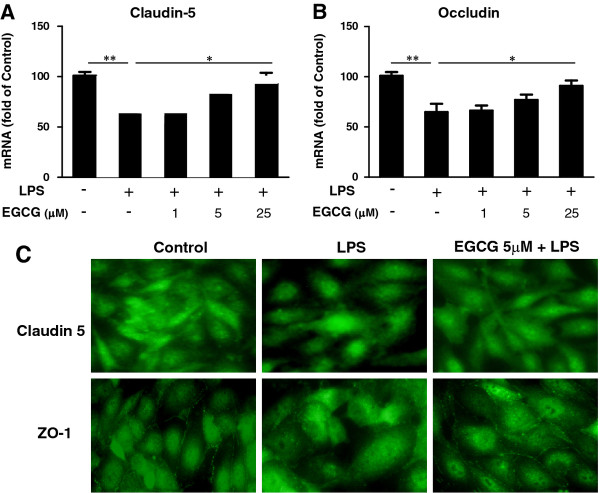
**Effect of (−)-epigallocatechin gallate on the expression of Claudin-5/Occludin of human cerebral microvascular endothelial cells/D3.** Human cerebral microvascular endothelial cells/D3 (hCMEC/D3) were pretreated with (−)-epigallocatechin gallate (EGCG) at the indicated doses for 1 h prior to lipopolysaccharide (LPS) treatment (100 ng/mL) for 24 h. RNA extracted was subjected to real time PCR for Claudin-5 and Occludin expression (**A**,**B**). Data are expressed as mean ± SD of three independent experiments. (**C**) hCMEC/D3 cells were pretreated with 5 μM EGCG for 1 h and then with 100 ng/ml LPS for 24 h. Cells were fixed and then incubated with anti-Claudin 5 or anti-ZO-1 antibody and observed under a fluorescence microscope (magnification **×** 200).

The tight junctional proteins, mainly Claudins and Occludins, are closely associated with the integrity of the BBB. We therefore examined the effect of EGCG on LPS-induced expression of these proteins. As shown in Figure 5A and B, LPS treatment suppressed the expression of Claudin 5 and Occludin in hCMEC/D3 cells at the mRNA level. This LPS effect, however, was inhibited in a dose-dependent fashion by EGCG pretreatment (Figure 5A, B). As it has been reported that Occludin was not consistently detected at cell-cell contacts of hCMEC/D3 cells [17], we only examined the protein expression of Claudin 5 and ZO-1 in hCMEC/D3 cells by immunofluorescence assay. Figure 5C and D shows that the LPS treatment compromised the expression of these two junctional proteins while EGCG pretreatment could protect the loss of Claudin 5 and ZO-1 at the cell-cell contacts.

### (−)-Epigallocatechin gallate protects blood–brain barrier permeability compromised by lipopolysaccharide treatment

**Figure 6:**
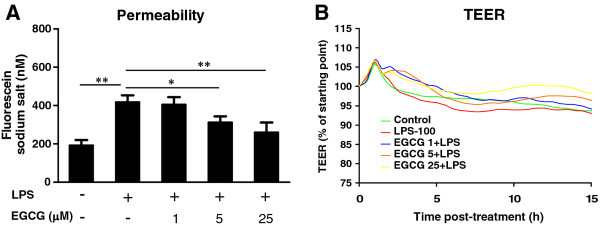
**Effect of (−)-epigallocatechin gallate on the permeability of human cerebral microvascular endothelial cells/D3.** (**A**) Human cerebral microvascular endothelial cells/D3 (hCMEC/D3) were cultured in 0.4-μm Millicell hanging cell culture inserts to become confluent and then treated with lipopolysaccharide (LPS) (100 ng/mL) in the presence or absence of (−)-epigallocatechin gallate (EGCG) at the indicated doses for 24 h. After treatment, fluorescein sodium salt was loaded to the apical filter compartment (1 μM) and the fluorescence in the basolateral compartment was measured within 1 h. (*P < 0.05, **P < 0.01). (**B**) hCMEC/D3 at 1 **×** 10^5^ per well were plated on collagen type I-coated 96W10E + electrode arrays (Applied Biophysics). The cells were then allowed to form monolayers reaching stable transendothelial electrical resistance (TEER) values. After 4 days (with a media change every 2 days), the monolayers were exposed to various concentrations of LPS with or without EGCG pretreatment at the indicated concentrations. The readings were acquired by the 1600R ECIS system continuously for 48 h at \00 Hz and at 30-minute intervals. Representative data from three independent experiments are shown.

We next examined the effect of LPS with or without EGCG pretreatment on the BBB permeability. As shown in Figure 6A, LPS treatment enhanced the diffusion of sodium fluorescein across the hCMEC/D3 monolayer to the basal compartment, while the pretreatment with EGCG reduced LPS-mediated diffusion of sodium fluorescein in the hCMEC/D3 cells (Figure 6A). hCMEC/D3 cells form a relative low TEER across the monolayer as compared with primary human brain microvascular endothelial cells [30]. Figure 6A shows that LPS treatment reduced the TEER of the hCMEC/D3 monolayer while EGCG pretreatment retains the TEER at a comparatively high level. Thus, EGCG has protective activity against LPS-induced compromise of BBB permeability.

### (−)-Epigallocatechin gallate inhibits lipopolysaccharide-induced NF-κB activation

**Figure 7:**
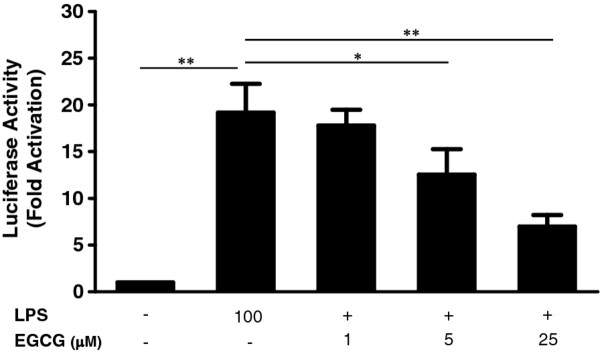
**Effect of (−)-epigallocatechin gallate on lipopolysaccharide-induced NF-**κ**B activation.** Human cerebral microvascular endothelial cells/D3 (hCMEC/D3) were transfected with pNF-κB-Luc plasmid and then treated with lipopolysaccharide (LPS) (1, 10, 100, or 1000 ng/mL) or (−)-epigallocatechin gallate (EGCG) (1, 5, or 25 μM) for 7 h. For pretreatment, cells were treated with EGCG (1, 5 or 25 μM) for 1 h prior to LPS treatment. Cells were lysed and the lysate was analyzed using a luciferase assay system. NF-κB promoter-driven luciferase activity was expressed as -fold of untreated cells (designated as 1). Data are expressed as mean ± SD of triplicates of two independent experiments. (*P < 0.05, **P < 0.01).

NF-κB is a key transcription factor for regulating the immune response to bacterial or viral infections. Dysregulations of NF-κB are associated with inflammatory and autoimmune diseases, viral infections and improper immune responses [31]. As shown in Figure 7, LPS treatment of hCMEC/D3 cells induced the activation of NF-κB. When the cells were pretreated with 25 μM EGCG, LPS-induced NF-κB activity was attenuated from 20-fold to 8-fold (Figure 7), while EGCG alone had little effect on NF-κB activation or inhibition (data not shown).

### 67-kDa laminin receptor is involved in (−)-epigallocatechin gallate-mediated suppression of lipopolysaccharide-mediated inflammatory cytokine induction

**Figure 8:**
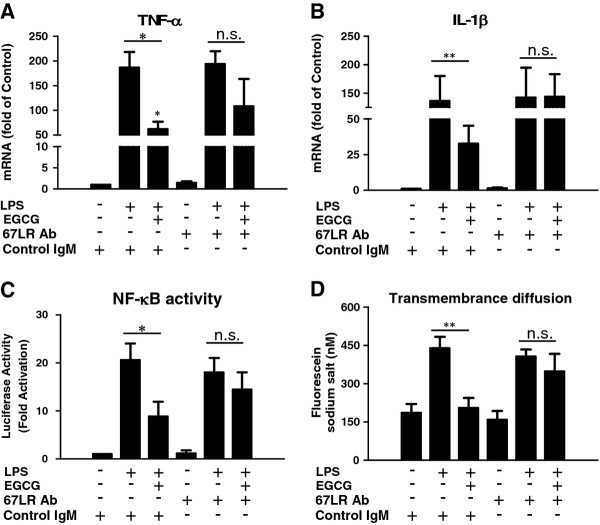
**Effect of 67-kDa laminin receptor neutralization on (−)-epigallocatechin gallate-mediated inhibition of lipopolysaccharide actions.** (**A**,**B**) Effect of 67-kDa laminin receptor (67LR) antibody on (−)-epigallocatechin gallate (EGCG)-mediated suppression of the induction of TNF-α and IL-1β by lipopolysaccharide (LPS). Human cerebral microvascular endothelial cells/D3 (hCMEC/D3) were treated with antibody against 67LR (5 μg/ml) or control IgM for 1 h prior to LPS and/or EGCG treatment. RNA extracted was subjected to real time PCR for TNF-α and IL-1β expression. (**C**) Effect of 67LR antibody on EGCG-mediated inhibition of the induction of NF-κB activity by LPS. Cells were transfected with pNF-κB-Luc plasmid and then treated with antibody against 67LR (5 μg/ml) or control IgM for 1 h prior to LPS and/or EGCG treatment for an additional 6 h. Luciferase activity was measured in the cell lysates. (**D**) Effect of 67LR antibody on EGCG-mediated suppression of transmembrane diffusion by LPS. The cells were treated with 67LR antibody, EGCG and LPS as described above. The apical apartment was then loaded with fluorescin sodium salt and the fluorescence in the basal apartment was measured within 1 h. Data are expressed as mean ± SD of three independent experiments. (*P < 0.05, **P < 0.01, compared with LPS treatment only).

**Figure 9:**
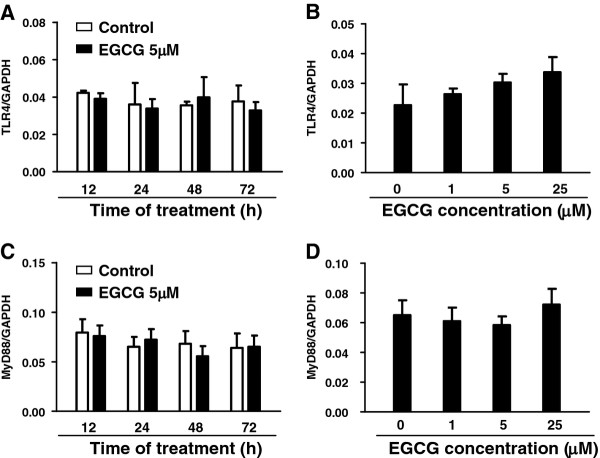
**Effect of (−)-epigallocatechin gallate on the TLR4 and MyD88 expression.** Human cerebral microvascular endothelial cells/D3 (hCMEC/D3) were treated with or without 5 μM of (−)-epigallocatechin gallate (EGCG) for the indicated time periods (**A**,**C**) or with EGCG at the indicated concentrations for 24 h (**B**,**D**). RNA extracted was subjected to real time PCR for TLR4 and MyD88 mRNA. Data are expressed as mean ± SD of three independent experiments.

67LR, a cell-surface receptor for EGCG, has been shown to be involved in the EGCG-mediated inhibitory effect on the TLR4 signaling pathway in macrophages [32]. We therefore examined whether EGCG acts through 67LR on the inhibition of LPS-mediated inflammation. We found that, when hCMEC/D3 cells were pretreated with neutralization antibody to 67LR prior to EGCG and LPS treatment, the inhibitory effect of EGCG on LPS-induced inflammatory cytokine expression was abrogated (Figure 8A,B). The blockage of 67LR also attenuated the inhibitory effect of EGCG on LPS-mediated NF-κB activation (Figure 8C) and increased fluorescein sodium transmembrance diffusion (Figure 8D). EGCG alone had little effect on the expression of TLR4 and MyD88 (Figure 9).

## Discussion

In the present study, we demonstrated that EGCG, a bioactive polyphenol in green tea, suppressed the expression of LPS-induced inflammatory cytokines in brain endothelial cells. This finding supports a previous report showing that EGCG attenuated LPS-mediated inflammation by suppressing the TNF-α and IL-1β expression in macrophages, leading to the downregulation of inflammatory responses [32].

EGCG pretreatment also inhibited the monocyte adhesion to the brain endothelial cell monolayer and blocked the negative impact of LPS on the tight junctional protein expression in brain endothelial cells. MCP-1/CCL2 plays a critical role in the recruitment of leukocytes to the site of inflammation elicited by LPS [33]. It has been reported that EGCG could decrease the MCP-1 and CCR2 gene expression, together with MCP-1 secretion and CCR2 expression at the cell surface in THP-1 monocytes, thus preventing the migration and adhesion of monocytes to fibronectin [34]. Our data showed that in unstimulated hCMEC/D3 cells, the basal expression of MCP-1/CCL2 was low but measurable, while LPS treatment greatly induced the expression of MCP-1/CCL2. This is consistent with a previous report [35] showing that hCMEC/D3 cells express lower levels of MCP-1/CCL2 than two primary human brain endothelial cells generated from multiple sclerosis brain tissue or from temporal lobe resections from epileptic patients.

In addition, we found that LPS treatment of hCMECs induced the expression of ICAMs and VCAMs, the key ligands for the beta2 integrin molecules present on leukocytes [36]. The expression of ICAM-1 in hCMEC/D3 cells is higher than that of VCAM-1 [17]. ICAM-1 and VCAM-1 facilitate the adhesion of monocytes to the endothelium. Endothelial ICAM-1 was shown to be essential for T cell diapedesis across the BBB *in vitro* under static conditions [37]. Adamson and colleagues [38] revealed that endothelial ICAM-1 was essentially involved in lymphocyte migration through brain endothelial cell monolayers by rearrangement of the endothelial actin cytoskeleton and functional endothelial cell Rho proteins. Treatment of HBMEC with TNF-α resulted in increased polymorphonuclear leukocyte adhesion that was significantly inhibited by blocking antibodies to E-selectin and ICAM-1, but not VCAM-1 [39].

It is well known that NF-κB activation is required for the LPS-induced inflammatory cytokine production [40]. Endothelial-selective blockade of NF-κB activation repressed expression of multiple endothelial adhesion molecules and reduced neutrophil infiltration into multiple organs [41]. EGCG was found to have the ability to block NF-κB activation in the intestinal epithelial cell line IEC-6 [42]. In addition, theaflavin-3,3’-digallate (another polyphenol) from black tea was also reported to have even stronger suppression of LPS-induced NF-κB activity than other polyphenols through downregulation of IκB kinase activity in macrophages [43]. Thus, the suppression of NF-κB activation by EGCG justifies the inhibitory effect of EGCG on LPS-mediated endothelial inflammation.

It is known that TLR4 is involved in LPS-mediated inflammation. Sheth and colleagues [44] showed that LPS disrupts tight junctions in cholangiocyte monolayers by a c-Scr-, TLR4- and LBP-dependent mechanism. A recent report [32] showed that EGCG downregulated inflammatory responses by directly suppressing TLR4 mRNA and protein expression. In addition, EGCG treatment of macrophages was found to upregulate the expression of Tollip [32], a negative regulator of the TLR signaling pathway. However, our data demonstrated that EGCG had little effect on TLR4 expression in brain endothelial cells. Youn and colleagues [45] reported that EGCG could inhibit LPS- or PolyI:C-mediated activation of interferon regulatory factor 3. EGCG could modulate both MyD88- and TIR-domain-containing adapter-inducing interferon-(TRIF)-dependent signaling pathways of TLR3 and the subsequent inflammatory target gene expression in macrophages. However, we did not observe a modulatory effect of EGCG on the expression of MyD88, a key adaptor to mediate the TLR-MyD88-dependent signaling pathway. These discrepancies may be due to the different types of cells used to investigate EGCG activity, suggesting that other mechanisms are likely to be involved in EGCG-mediated inhibition of endothelial inflammation.

67LR is a receptor that presents on the eukaryotic cell membrane for the cellular prion proteins and also interacts with extracellular matrix components [46]. 67LR was also found to express on both rodent and human cerebral endothelial cells [47]. It has been suggested that 67LR serves as a co-receptor for bacterial pathogens that target the BBB. 67LR on brain endothelial cells interacts with three most commonly neuroinvasive bacteria: pneumococcus, *H. influenzae*, and meningococcus. The binding of 67LR to these pathogens initiates the bacterial interaction with the BBB and promote their CNS tropism, inducing cell signaling through the other receptors, such as the TLRs. The interaction of TLRs with 67LR may synergistically promote bacterial adherence and invasion of BBB [47]. Interestingly, 67LR was shown to be involved in the inhibitory effect of EGCG on the TLR4 signaling pathway in macrophages [32]. These findings promoted our interest in examining the role of 67LR in the anti-inflammatory effect of EGCG in brain endothelial cells. Our data that pretreatment of hCMEC cells with anti-67LR antibody significantly blocked the EGCG effect on LPS-mediated TNF-α and IL-1β induction, as well as on NF-κB activation, indicate that EGCG exerts its anti-inflammatory effect in endothelial cells at least partially through 67LR. It is likely that pretreatment of hCMEC/D3 cells with EGCG enables the binding of 67LR to EGCG and disrupts or modulates LPS interaction with 67LR. It has been suggested this disruption or modulation might engender unexpectedly broad protection against systemic infections [47]. Thus, our data support the notion that EGCG can be used as a potential therapeutic compound to treat CNS inflammation related to the BBB.

## Abbreviations

67-LR, 67-kDa laminin receptor; ANOVA, analysis of variance; BBB, blood–brain barrier; CNS, central nervous system; DMEM, Dulbecco’s modeified Eagle’s medium; EGCG, (−)-epigallocatechin gallate; ELISA, enzyme-linked immunosorbent assay; GAPDH, glyceraldehyde 3-phosphate dehydrogenase; HBMEC, human brain microvascular endothelial cells; hCMEC, human cerebral microvascular endothelial cell; ICAM, intercellular adhesion molecule; IL, interleukin; iNOS, inducible nitric oxide synthase; LPS, lipopolysaccharide; MCP, monocyte chemotactic protein; NF, nuclear factor; PBS, phosphate-buffered saline; PMN, polymorphonuclear leukocytes; qRT-PCR, quantitative real time polymerase chain reaction; TEER, transendothelial electrical resistance; TLR, Toll-like receptor; TNF, tumor necrosis factor; VCAM, vascular adhesion molecule.

## Competing interests

The authors declare that they have no competing interests.

## Authors' contributions

JL and LY designed and performed experiments, and drafted the manuscript. JL, LY, XW, YW and YZ performed experiments. WZH conceived of the study, participated in its design and coordination, and drafted the manuscript. All authors have read and approved the final version of this manuscript.

## References

[B1] Marshall JC Endotoxin in the pathogenesis of sepsis Contrib Nephrol 2010 167 1 13 20519894 10.1159/000315914

[B2] Yoneda O Imai T Goda S Inoue H Yamauchi A Okazaki T Imai H Yoshie O Bloom ET Domae N Umehara H Fractalkine-mediated endothelial cell injury by NK cells J Immunol 2000 164 4055 4062 10754298 10.4049/jimmunol.164.8.4055

[B3] Zhang WJ Wei H Hagen T Frei B Alpha-lipoic acid attenuates LPS-induced inflammatory responses by activating the phosphoinositide 3-kinase/Akt signaling pathway Proc Natl Acad Sci U S A 2007 104 4077 4082 10.1073/pnas.0700305104 17360480 PMC1805485

[B4] Freyer D Manz R Ziegenhorn A Weih M Angstwurm K Docke WD Meisel A Schumann RR Schonfelder G Dirnagl U Weber JR Cerebral endothelial cells release TNF-alpha after stimulation with cell walls of Streptococcus pneumoniae and regulate inducible nitric oxide synthase and ICAM-1 expression via autocrine loops J Immunol 1999 163 4308 4314 10510370

[B5] Corsini E Dufour A Ciusani E Gelati M Frigerio S Gritti A Cajola L Mancardi GL Massa G Salmaggi A Human brain endothelial cells and astrocytes produce IL-1 beta but not IL-10 Scand J Immunol 1996 44 506 511 10.1046/j.1365-3083.1996.d01-343.x 8947603

[B6] Verma S Nakaoke R Dohgu S Banks WA Release of cytokines by brain endothelial cells: a polarized response to lipopolysaccharide Brain Behav Immun 2006 20 449 455 10.1016/j.bbi.2005.10.005 16309883

[B7] Dohgu S Fleegal-DeMotta MA Banks WA Lipopolysaccharide-enhanced transcellular transport of HIV-1 across the blood–brain barrier is mediated by luminal microvessel IL-6 and GM-CSF J Neuroinflammation 2011 8 167 10.1186/1742-2094-8-167 22129063 PMC3260201

[B8] Chui R Dorovini-Zis K Regulation of CCL2 and CCL3 expression in human brain endothelial cells by cytokines and lipopolysaccharide J Neuroinflammation 2010 7 1 10.1186/1742-2094-7-1 20047691 PMC2819252

[B9] Amos C Romero IA Schultze C Rousell J Pearson JD Greenwood J Adamson P Cross-linking of brain endothelial intercellular adhesion molecule (ICAM)-1 induces association of ICAM-1 with detergent-insoluble cytoskeletal fraction Arterioscler Thromb Vasc Biol 2001 21 810 816 10.1161/01.ATV.21.5.810 11348879

[B10] Wong D Dorovini-Zis K Upregulation of intercellular adhesion molecule-1 (ICAM-1) expression in primary cultures of human brain microvessel endothelial cells by cytokines and lipopolysaccharide J Neuroimmunol 1992 39 11 21 10.1016/0165-5728(92)90170-P 1352310

[B11] Frigerio S Gelati M Ciusani E Corsini E Dufour A Massa G Salmaggi A Immunocompetence of human microvascular brain endothelial cells: cytokine regulation of IL-1beta, MCP-1, IL-10, sICAM-1 and sVCAM-1 J Neurol 1998 245 727 730 10.1007/s004150050275 9808241

[B12] Wu CC Hsu MC Hsieh CW Lin JB Lai PH Wung BS Upregulation of heme oxygenase-1 by epigallocatechin-3-gallate via the phosphatidylinositol 3-kinase/Akt and ERK pathways Life Sci 2006 78 2889 2897 10.1016/j.lfs.2005.11.013 16378625

[B13] Rezai-Zadeh K Arendash GW Hou H Fernandez F Jensen M Runfeldt M Shytle RD Tan J Green tea epigallocatechin-3-gallate (EGCG) reduces beta-amyloid mediated cognitive impairment and modulates tau pathology in Alzheimer transgenic mice Brain Res 2008 1214 177 187 18457818 10.1016/j.brainres.2008.02.107

[B14] Ahn HY Xu Y Davidge ST Epigallocatechin-3-O-gallate inhibits TNFalpha-induced monocyte chemotactic protein-1 production from vascular endothelial cells Life Sci 2008 82 964 968 10.1016/j.lfs.2008.02.018 18397796

[B15] Lee H Bae JH Lee SR Protective effect of green tea polyphenol EGCG against neuronal damage and brain edema after unilateral cerebral ischemia in gerbils J Neurosci Res 2004 77 892 900 10.1002/jnr.20193 15334607

[B16] Sutherland BA Shaw OM Clarkson AN Jackson DN Sammut IA Appleton I Neuroprotective effects of (−)-epigallocatechin gallate following hypoxia-ischemia-induced brain damage: novel mechanisms of action FASEB J 2005 19 258 260 15569775 10.1096/fj.04-2806fje

[B17] Weksler BB Subileau EA Perrière N Charneau P Holloway K Leveque M Tricoire-Leignel H Nicotra A Bourdoulous S Turowski P Male DK Roux F Greenwood J Romero IA Couraud PO Blood–brain barrier-specific properties of a human adult brain endothelial cell line FASEB J 2005 19 1872 1874 16141364 10.1096/fj.04-3458fje

[B18] Cucullo L Couraud PO Weksler B Romero IA Hossain M Rapp E Janigro D Immortalized human brain endothelial cells and flow-based vascular modeling: a marriage of convenience for rational neurovascular studies J Cereb Blood Flow Metab 2008 28 312 328 10.1038/sj.jcbfm.9600525 17609686

[B19] Afonso PV Ozden S Cumont MC Seilhean D Cartier L Rezaie P Mason S Lambert S Huerre M Gessain A Couraud PO Pique C Ceccaldi PE Romero IA Alteration of blood–brain barrier integrity by retroviral infection PLoS Pathog 2008 4 e1000205 10.1371/journal.ppat.1000205 19008946 PMC2575404

[B20] Vu K Weksler B Romero I Couraud PO Gelli A Immortalized human brain endothelial cell line HCMEC/D3 as a model of the blood–brain barrier facilitates in vitro studies of central nervous system infection by Cryptococcus neoformans Eukaryot Cell 2009 8 1803 1807 10.1128/EC.00240-09 19767445 PMC2772405

[B21] Poller B Gutmann H Krahenbuhl S Weksler B Romero I Couraud PO Tuffin G Drewe J Huwyler J The human brain endothelial cell line hCMEC/D3 as a human blood–brain barrier model for drug transport studies J Neurochem 2008 107 1358 1368 10.1111/j.1471-4159.2008.05730.x 19013850

[B22] Li J Hu S Zhou L Ye L Wang X Ho J Ho W Interferon lambda inhibits herpes simplex virus type I infection of human astrocytes and neurons Glia 2011 59 58 67 10.1002/glia.21076 20878770 PMC3082435

[B23] Li J Ye L Cook DR Wang X Liu J Kolson DL Persidsky Y Ho WZ Soybean-derived Bowman-Birk inhibitor inhibits neurotoxicity of LPS-activated macrophages J Neuroinflammation 2011 8 15 10.1186/1742-2094-8-15 21324129 PMC3046894

[B24] Ramirez SH Fan S Dykstra H Reichenbach N Del Valle L Potula R Phipps RP Maggirwar SB Persidsky Y Dyad of CD40/CD40 ligand fosters neuroinflammation at the blood–brain barrier and is regulated via JNK signaling: implications for HIV-1 encephalitis J Neurosci 2010 30 9454 9464 20631174 10.1523/JNEUROSCI.5796-09.2010 PMC2908988

[B25] Weidenfeller C Svendsen CN Shusta EV Differentiating embryonic neural progenitor cells induce blood–brain barrier properties J Neurochem 2007 101 555 565 10.1111/j.1471-4159.2006.04394.x 17254017 PMC2657050

[B26] Argaw AT Gurfein BT Zhang Y Zameer A John GR VEGF-mediated disruption of endothelial CLN-5 promotes blood–brain barrier breakdown Proc Natl Acad Sci U S A 2009 106 1977 1982 10.1073/pnas.0808698106 19174516 PMC2644149

[B27] Petrak D Memon SA Birrer MJ Ashwell JD Zacharchuk CM Dominant negative mutant of c-Jun inhibits NF-AT transcriptional activity and prevents IL-2 gene transcription J Immunol 1994 153 2046 2051 8051409

[B28] Pierce JW Lenardo M Baltimore D Oligonucleotide that binds nuclear factor NF-kappa B acts as a lymphoid-specific and inducible enhancer element Proc Natl Acad Sci U S A 1988 85 1482 1486 10.1073/pnas.85.5.1482 3125549 PMC279795

[B29] Luckow B Schutz G CAT constructions with multiple unique restriction sites for the functional analysis of eukaryotic promoters and regulatory elements Nucleic Acids Res 1987 15 5490 10.1093/nar/15.13.5490 3037497 PMC305985

[B30] Ramirez SH Haskó J Skuba A Fan S Dykstra H McCormick R Reichenbach N Krizbai I Mahadevan A Zhang M Tuma R Son YJ Persidsky Y Activation of cannabinoid receptor 2 attenuates leukocyte-endothelial cell interactions and blood–brain barrier dysfunction under inflammatory conditions J Neurosci 2012 32 4004 4016 10.1523/JNEUROSCI.4628-11.2012 22442067 PMC3325902

[B31] Giuliani C Napolitano G Bucci I Montani V Monaco F Nf-kB transcription factor: role in the pathogenesis of inflammatory, autoimmune, and neoplastic diseases and therapy implications Clin Ter 2001 152 249 253 11725618

[B32] Hong Byun E Fujimura Y Yamada K Tachibana H TLR4 signaling inhibitory pathway induced by green tea polyphenol epigallocatechin-3-gallate through 67-kDa laminin receptor J Immunol 2010 185 33 45 10.4049/jimmunol.0903742 20511545

[B33] Strieter RM Belperio JA Keane MP Cytokines in innate host defense in the lung J Clin Invest 2002 109 699 705 11901175 10.1172/JCI15277 PMC150916

[B34] Melgarejo E Medina MA Sanchez-Jimenez F Urdiales JL Epigallocatechin gallate reduces human monocyte mobility and adhesion in vitro Br J Pharmacol 2009 158 1705 1712 10.1111/j.1476-5381.2009.00452.x 19912233 PMC2801211

[B35] Subileau EA Rezaie P Davies HA Colyer FM Greenwood J Male DK Romero IA Expression of chemokines and their receptors by human brain endothelium: implications for multiple sclerosis J Neuropathol Exp Neurol 2009 68 227 240 10.1097/NEN.0b013e318197eca7 19225413

[B36] Foy DS Ley K Intercellular adhesion molecule-1 is required for chemoattractant-induced leukocyte adhesion in resting, but not inflamed, venules in vivo Microvasc Res 2000 60 249 260 10.1006/mvre.2000.2272 11078641

[B37] Steiner O Coisne C Cecchelli R Boscacci R Deutsch U Engelhardt B Lyck R Differential roles for endothelial ICAM-1, ICAM-2, and VCAM-1 in shear-resistant T cell arrest, polarization, and directed crawling on blood–brain barrier endothelium J Immunol 2010 185 4846 4855 10.4049/jimmunol.0903732 20861356

[B38] Adamson P Etienne S Couraud PO Calder V Greenwood J Lymphocyte migration through brain endothelial cell monolayers involves signaling through endothelial ICAM-1 via a rho-dependent pathway J Immunol 1999 162 2964 2973 10072547

[B39] Wong D Prameya R Dorovini-Zis K Adhesion and migration of polymorphonuclear leukocytes across human brain microvessel endothelial cells are differentially regulated by endothelial cell adhesion molecules and modulate monolayer permeability J Neuroimmunol 2007 184 136 148 10.1016/j.jneuroim.2006.12.003 17291598

[B40] Mandrekar P Catalano D Szabo G Inhibition of lipopolysaccharide-mediated NFkappaB activation by ethanol in human monocytes Int Immunol 1999 11 1781 1790 10.1093/intimm/11.11.1781 10545482

[B41] Ye X Ding J Zhou X Chen G Liu SF Divergent roles of endothelial NF-kappaB in multiple organ injury and bacterial clearance in mouse models of sepsis J Exp Med 2008 205 1303 1315 10.1084/jem.20071393 18474628 PMC2413029

[B42] Yang F Oz HS Barve S de Villiers WJ McClain CJ Varilek GW The green tea polyphenol (−)-epigallocatechin-3-gallate blocks nuclear factor-kappa B activation by inhibiting I kappa B kinase activity in the intestinal epithelial cell line IEC-6 Mol Pharmacol 2001 60 528 533 11502884

[B43] Pan MH Lin-Shiau SY Ho CT Lin JH Lin JK Suppression of lipopolysaccharide-induced nuclear factor-kappaB activity by theaflavin-3,3'-digallate from black tea and other polyphenols through down-regulation of IkappaB kinase activity in macrophages Biochem Pharmacol 2000 59 357 367 10.1016/S0006-2952(99)00335-4 10644043

[B44] Sheth P Delos Santos N, Seth A, LaRusso NF, Rao RK: Lipopolysaccharide disrupts tight junctions in cholangiocyte monolayers by a c-Src-, TLR4-, and LBP-dependent mechanism Am J Physiol Gastrointest Liver Physiol 2007 293 G308 318 10.1152/ajpgi.00582.2006 17446308

[B45] Youn HS Lee JY Saitoh SI Miyake K Kang KW Choi YJ Hwang DH Suppression of MyD88- and TRIF-dependent signaling pathways of Toll-like receptor by (−)-epigallocatechin-3-gallate, a polyphenol component of green tea Biochem Pharmacol 2006 72 850 859 10.1016/j.bcp.2006.06.021 16890209

[B46] Kim KJ Chung JW Kim KS 67-kDa laminin receptor promotes internalization of cytotoxic necrotizing factor 1-expressing Escherichia coli K1 into human brain microvascular endothelial cells J Biol Chem 2005 280 1360 1368 15516338 10.1074/jbc.M410176200

[B47] Orihuela CJ Mahdavi J Thornton J Mann B Wooldridge KG Abouseada N Oldfield NJ Self T Ala'Aldeen DA, Tuomanen EI: Laminin receptor initiates bacterial contact with the blood brain barrier in experimental meningitis models J Clin Invest 2009 119 1638 1646 10.1172/JCI36759 19436113 PMC2689107

